# Redescription and molecular analysis of *Neoechinorhynchus (Neoechinorhynchus) johnii* Yamaguti, 1939 (Acanthocephala, Neoechinorhynchidae) from the Pacific Ocean off Vietnam

**DOI:** 10.1051/parasite/2019041

**Published:** 2019-07-23

**Authors:** Omar M. Amin, Anshu Chaudhary, Richard Heckmann, Nguyen V. Ha, Hridaya S. Singh

**Affiliations:** 1 Institute of Parasitic Diseases 11445 E. Via Linda 2-419 Scottsdale AZ 85259 USA; 2 Molecular Taxonomy Laboratory, Department of Zoology, Chaudhary Charan Singh University Meerut Uttar Pradesh 250004 India; 3 Department of Biology, Brigham Young University 1114 MLBM Provo UT 84602 USA; 4 Department of Parasitology, Institute of Ecology and Biological Resources (IEBR), Vietnam Academy of Science and Technology 18 Hoang Quoc Viet Cau Giay Hanoi Vietnam

**Keywords:** Acanthocephala, *Neoechinorhynchus johnii*, Diversity, Molecular description, Hosts and distribution

## Abstract

*Neoechinorhynchus* (*Neoechinorhynchus*) *johnii* Yamaguti, 1939 is redescribed from *Eleutheronema tetradactylum* (Polynemidae), *Johnius carouna* (Sciaenidae), *Johnius* sp., and *Otolithes ruber* (Sciaenidae) along the north and south coasts of Vietnam. Our description completes missing and inadequate information in the original descriptions and line drawings from *Johnius goma* in Japan and from *Pseudosciaena diacanthus* in the Indian Ocean. We add new information documented by scanning electron microscopy (SEM) and photomicroscopy, and explore the wide morphological diversity attributed to host species. The redescription includes: worms cylindrical with round proboscis with prominent apical organ, and large anterior hooks distant from small middle and posterior hooks; neck longer than the proboscis, nucleated lemnisci subequal, and receptacle with large basal triangulate cephalic ganglion and attached para-receptacle structure (PRS); male reproductive system in posterior half of trunk; adult females with introvert genital vestibule; and eggs spherical or rectangular. Gallium cuts and X-ray scans of hooks show high concentrations of sulfur on edge layer aiding in forming hardened calcium phosphate apatite of that layer with calcium and phosphorus in higher concentration in central part of hook. Molecular results consistently yielded a strongly supported distinct clade for the *Neoechinorhynchus* species from Vietnam for both 18S gene and the ITS1-5.8S-ITS2 region of ribosomal RNA. Phylogenetic analysis demonstrated that *N. johnii* occupies a separate position in the trees, probably indicating an Asian origin of this species.

## Abstract (fr)

*Neoechinorhynchus (Neoechinorhynchus) johnii* Yamaguti, 1939 est redécrit de *Eleutheronema tetradactylum* (Polynemidae), *Johnius carouna* (Sciaenidae), *Johnius* sp. et *Otolithes ruber* (Sciaenidae) des côtes nord et sud du Viêt Nam. Notre description complète les informations manquantes et inadéquates dans les descriptions originales et les dessins au trait de spécimens de *Johnius goma* au Japon et de *Pseudosciaena diacanthus* de l’océan Indien. Nous ajoutons de nouvelles informations par microscopie électronique à balayage et photomicroscopie, et explorons la grande diversité morphologique attribuée aux espèces hôtes. La nouvelle description comprend : vers cylindriques à trompe arrondie avec un organe apical proéminent et de grands crochets antérieurs éloignés des petits crochets médians et postérieurs ; cou plus long que le proboscis, lemnisques nucléés subégaux, et réceptacle avec un grand ganglion céphalique triangulaire basal et une structure de para-réceptacle attachée ; système reproducteur mâle dans la moitié postérieure du tronc ; femelles adultes avec vestibule génital introverti ; oeufs sphériques ou rectangulaires. Les coupes au gallium et les analyses aux rayons X des crochets montrent de fortes concentrations de soufre sur la couche marginale, contribuant à la formation d’apatite de phosphate de calcium durci de cette couche, avec une concentration plus élevée de calcium et de phosphore dans la partie centrale du crochet. Les résultats moléculaires pour le gène 18S et les régions ITS1-5.8S-ITS2 de l’ARN ribosomal ont régulièrement montré un clade distinct et fortement soutenu pour les espèces de *Neoechinorhynchus* du Viêt Nam. L’analyse phylogénétique a démontré que *N. johnii* occupe une position distincte dans les arbres, indiquant probablement une origine asiatique de cette espèce.

## Introduction

Most of the recent taxonomic work on the Acanthocephala from Vietnam was reported by the Amin-Heckmann-Ha team since 2000. A number of acanthocephalan species from freshwater fish, amphibians, reptiles, birds, and mammals were previously described in Vietnam [5, 10–14]. Additionally, 11 species of acanthocephalans were collected from marine fish off the eastern seaboard of Vietnam in Halong Bay in 2008 and 2009. Of these, six new species of *Neoechinorhynchus* Stiles & Hassall 1905, one new species of *Heterosentis* Van Cleave, 1931, and two new species of *Rhadinorhynchus* Lühe 1911 were described [9, 15, 16]. Four other species of Echinorhynchid acanthocephalans from marine fishes in Halong Bay were also described [6], and five other new species from fishes and amphibians of eight collected host species were described [18]. Three other species of *Rhadinorhynchus* and one species of *Gorgorhynchus* were otherwise previously reported from marine fishes in Vietnam [20].

Fifteen species of acanthocephalans in five families were more recently collected from fishes in the Pacific and amphibians in central Vietnam in 2016 and 2017. The revision of the genus *Neoechinorhynchus* and the erection of two subgenera [2, 3] complement the above reports to produce the present account redescribing *N. johnii* using SEM and photomicroscopy. We also report new features including results of metal analysis of hooks (energy dispersive analysis for X-ray, EDAX) and expand the host and geographical distribution of the species. We also include the molecular description of *N. johnii* Yamaguti, 1939 and describe its phylogenetic relationships.

## Materials and methods

### Collections

Specimens were collected using gill nets from research vessels off shore at noted collection sites. Information on the collections of specimens of *N. johnii* is presented in Table 1.

**Table 1:** Host and geographical distribution of *Neoechinorhynchus johnii* in the Pacific Ocean off Vietnam.

Hosts	Specimens	Date	Location	Coordinates
*Eleutheronema tetradactylus* (Shaw)	3♀♀ in 1/1 fish	2016	Vung Tau	10°23′N, 107°7′E
	4♂♂, 8♀♀ in 3/12 fish	July, 2017	Bac Lieu	9°15′N, 105°45′E
*Johnius carouna* (Cuvier)	11♂♂, 14♀♀	Oct., 2017	Nha Trang	12°15′N, 109°11′E
	5♀♀ in 1/10 fish	July, 2017	Bac Lieu	9°15′N, 105°45′E
*Johnius* sp.	1♂, 1♀ in 1 fish	March, 2017	Tien Yen	21°20′N, 107°24′E
*Otolithes ruber* (Bloch & Schneider)	3♂♂, 5♀♀	May, 2017	Quang Binh	17°30′N, 106°20′E
Total	20♂♂, 40♀♀			

### Processing for microscopical studies

Freshly collected acanthocephalans were extended in water until proboscides were everted, and fixed in 70% ethanol for transport to our Institute of Parasitic Diseases (IPD) in Arizona, USA for processing and further studies. Worms were punctured with a fine needle and subsequently stained in Mayer’s acid carmine, destained in 4% hydrochloric acid in 70% ethanol, dehydrated in ascending concentrations of ethanol reaching 100% (24 h each), and cleared in 100% xylene then in 50% Canada balsam and 50% xylene (24 h each). Whole worms were then mounted in Canada balsam. Measurements are in micrometers, unless otherwise noted; the range is followed by the mean values between parentheses. Width measurements represent maximum width. Trunk length does not include proboscis, neck, or bursa.

Voucher specimens were deposited in the University of Nebraska’s State Museum’s Harold W. Manter Laboratory (HWML) collection in Lincoln, Nebraska, USA.

### Scanning electron microscopy (SEM)

Specimens that had been fixed and stored in 70% ethanol were processed for SEM following standard methods [29]. These included critical point drying (CPD) in sample baskets and mounting on SEM sample mounts (stubs) using conductive double-sided carbon tape. Samples were coated with gold and palladium for 3 min using a Polaron #3500 sputter coater (Quorum (Q150 TES) https://www.quorumtech.com) establishing an approximate thickness of 20 nm. Samples were placed and observed in an FEI Helios Dual Beam Nanolab 600 (FEI, Hillsboro, Oregon) Scanning Electron Microscope, with digital images obtained in the Nanolab software system (FEI, Hillsboro, Oregon), and then transferred to a USB for future reference. Samples were received under low vacuum conditions using 10 kV, spot size 2, 0.7 Torr using a GSE detector.

### X-ray microanalysis using energy dispersive analysis for X-ray (EDAX)

Standard methods were used for preparation, similar to the SEM procedure. Specimens were examined and positioned with the above SEM instrument which was equipped with a Phoenix energy-dispersive X-ray analyzer (FEI, Hillsboro, Oregon). X-ray spot analysis and live scan analysis were performed at 16 kV with a spot size of five and results were recorded on charts and stored with digital imaging software attached to a computer. The TEAM* (Texture and Elemental Analytical Microscopy) software system (FEI, Hillsboro, Oregon) was used. Data were stored on a USB for future analysis. The data included weight percent and atom percent of the detected elements following correction factors.

### Ion sectioning of hooks

A dual-beam SEM with a gallium (Ga) ion source (GIS) was used for the liquid ion metal source (LIMS) part of the process. The hooks of the acanthocephalans were centered on the SEM stage and cross-sectioned using a probe current between 0.2 nA and 2.1 nA according to the rate at which the area was cut. The time of cutting was based on the nature and sensitivity of the tissue. Following the initial cut, the sample also went through a milling process to obtain a smooth surface. The cut was then analyzed with X-ray at the tip, middle, and base of hooks for chemical ions with an electron beam (Tungsten) to obtain an X-ray spectrum. Results were stored with the attached imaging software then transferred to a USB for future use. The intensity of the GIS was variable, according to the nature of the material being cut.

### Molecular methods

Genomic DNA of the worms preserved in 95% ethanol was extracted individually using a DNeasy™ Blood and Tissue kit (Qiagen, Germany), according to the manufacturer’s instructions. Isolated DNA was amplified by PCR using the primer pairs: Worm A (5′-GCGAATGGCTCATTAAATCAG-3′), 270R (5′-CCGTCAATTCCTTTAAGT-3′) [30] for the 18S gene; and BD1 (5′-GTCGTAACAAGGTTTCCGTA-3′) and BD2 (5′-TATGCTTAAATTCAGCGGGT-3′) [23] for the ITS1+5.8S+ITS2 region. Amplification of the PCR reactions was carried in a 25 μL reaction mixture containing 3 μL DNA, 2.5 μL of 10 X Taq buffer (Biotools, Madrid, Spain), 1 μL of Taq polymerase (1 U, Biotools), 3 μL of deoxyribonucleoside triphosphates, 1 μL of each forward and reverse primer, and 13.5 μL of water. PCR cycling parameters comprised an initial denaturation at 94 °C for 3 min, followed by 40 cycles of 94 °C for 45 s, 55 °C for 45 s, 72 °C for 1 min, and subsequent final elongation at 72 °C for 10 min, then stored at 4 °C. Amplification products were electrophoresed through 1% agarose gel in TAE buffer and examined under ultraviolet light. The amplified DNA was then purified with the Purelink™ Quick Gel Extraction and PCR Purification Combo Kit (Invitrogen). Obtained amplification products were sequenced with the Big Dye Terminator v. 3.1 cycle sequencing kit in an ABI 3130 Genetic Analyzer, Applied Biosystems, using the above-mentioned primers.

Alignment and analysis of sequences obtained during the study were carried out using BioEdit, version 7.2.5 [26]. Sequences of 18S and the ITS1+5.8S+ITS2 region from closely related acanthocephalans were determined by BLASTn search and downloaded for further analysis.

Multiple sequences alignment was performed using ClustalW as implemented in MEGA v. 6.0 [36] together with other species of acanthocephalans. Phylogenetic analyses were conducted using MEGA v. 6.0 [36] with 1000 bootstrap replicates for prior testing of reliability and Bayesian inference (BI) analyses in Topali 2.5 [31]. For nucleotide alignment of 18S and the ITS1+5.8S+ITS2 region, a phylogenetic tree was constructed using the maximum likelihood (ML) method in MEGA v. 6.0. Pairwise distance analyses were carried out using the Maximum Composite Likelihood model [36]. For Bayesian inference (BI) analyses, the substitution models were tested by the Bayesian Information Criterion and GTR+G+I was chosen. Posterior probabilities were estimated over 1,000,000 generations via five independent runs of four simultaneous MCMCMC chains with every 100th tree saved. The “burn in” was set to 25%.

## Results

### *Neoechinorhynchus* (*Neoechinorhynchus*) *johnii* Yamaguti, 1939

Family: Neoechinorhynchidae

Genus: *Neoechinorhynchus*

Subgenus: *Neoechinorhynchus*

Hosts, localities, and dates: See Table 1.

Site of infection: Intestine.

Specimens: HWML collection Nos. 139459, 139460, 139465, 139466, 139468 (from *J. carouna*), 139461, 139463, 139464 (from *E. tetradactylum*), 139462, 139467 (from *O. ruber*).

Representative DNA sequences: The 18S and ITS1-5.8S-ITS2 region of rDNA sequences of *N*. *johnii* were deposited in GenBank under the accession numbers MK260005 and MK260007 (for the 18S gene), and MK260006 and MK260008 (for the ITS region).

### Morphological description

**Figures 1–6:**
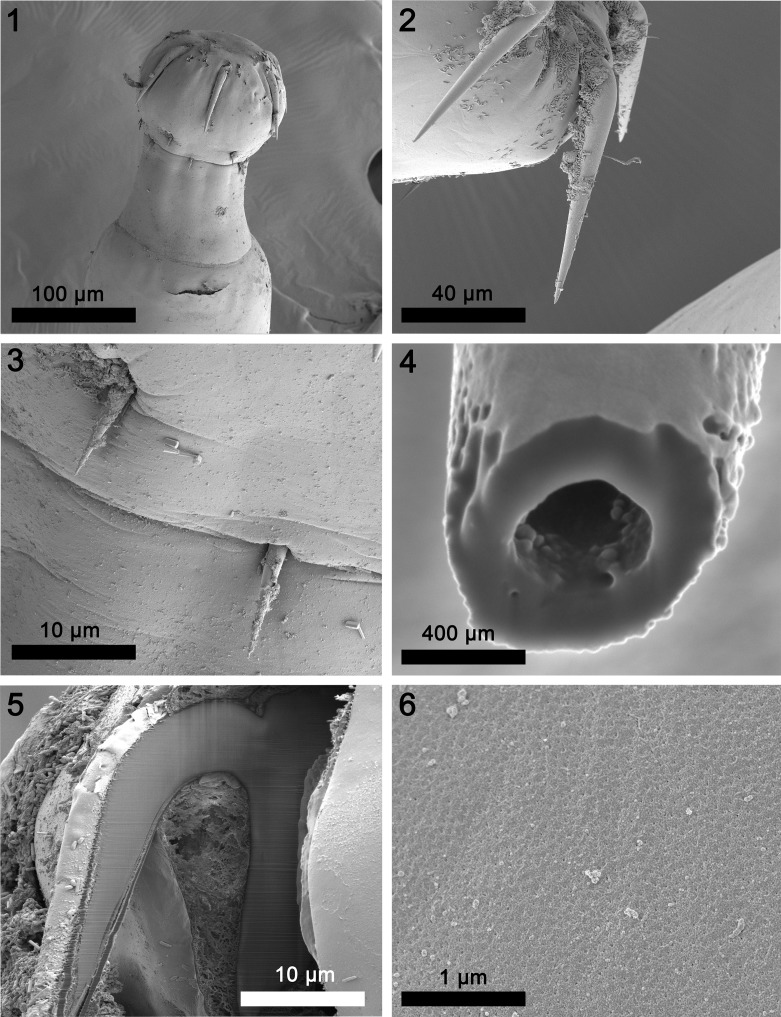
SEM of specimens of *Neoechinorhynchus johnii* from *Eleutheronema tetradactylus, Johnius carouna,* and *Otolithes ruber* in the Pacific Ocean off Vietnam. (1) The proboscis and neck of a male specimen. (2) Two anterior proboscis hooks of another specimen. Note the curvature at the base of the otherwise straight hook. (3) Middle and posterior hooks in the proboscis in Figure 1. Note the location of the posterior hook at the furrow separating the proboscis from the neck. (4) A gallium cut cross-section of a hook showing its vacuolated hollow core. (5) A gallium cut longitudinal section near the edge of an anterior hook showing the distinction between the thin cortical layer and the dense core. Note the prominent posteriorly directed root and the slightly manubriated anterior end. (6) Micropores at the mid trunk of a male specimen.

**Figures 7–12:**
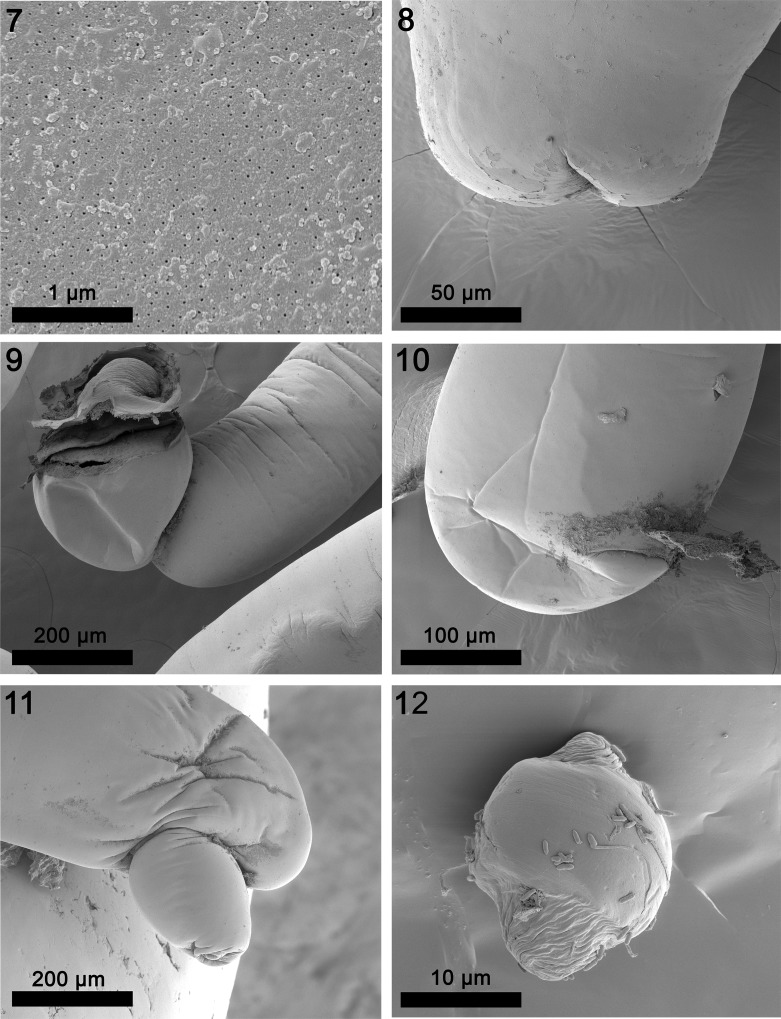
SEM of specimens of *Neoechinorhynchus johnii* from *Eleutheronema tetradactylus, Johnius carouna,* and *Otolithes ruber* in the Pacific Ocean off Vietnam. (7) Micropores at the posterior trunk of the same male specimen in Figure 6. Note the different pore diameter and distribution indicative of differential nutrient absorption of different parts of the trunk. (8) The posterior end of a male specimen showing the site of the invaginated bursa. (9) The plain evaginated bursa tilting ventral at the posterior end of a male specimen. (10) Normal rounded outline of the posterior end of a female specimen. (11) A constriction occasionally seen at the posterior end of female specimens contains the looped posterior end of the reproductive system. (12) An egg of the lantern-like variety with fibrous elements.

**Figures 13–20:**
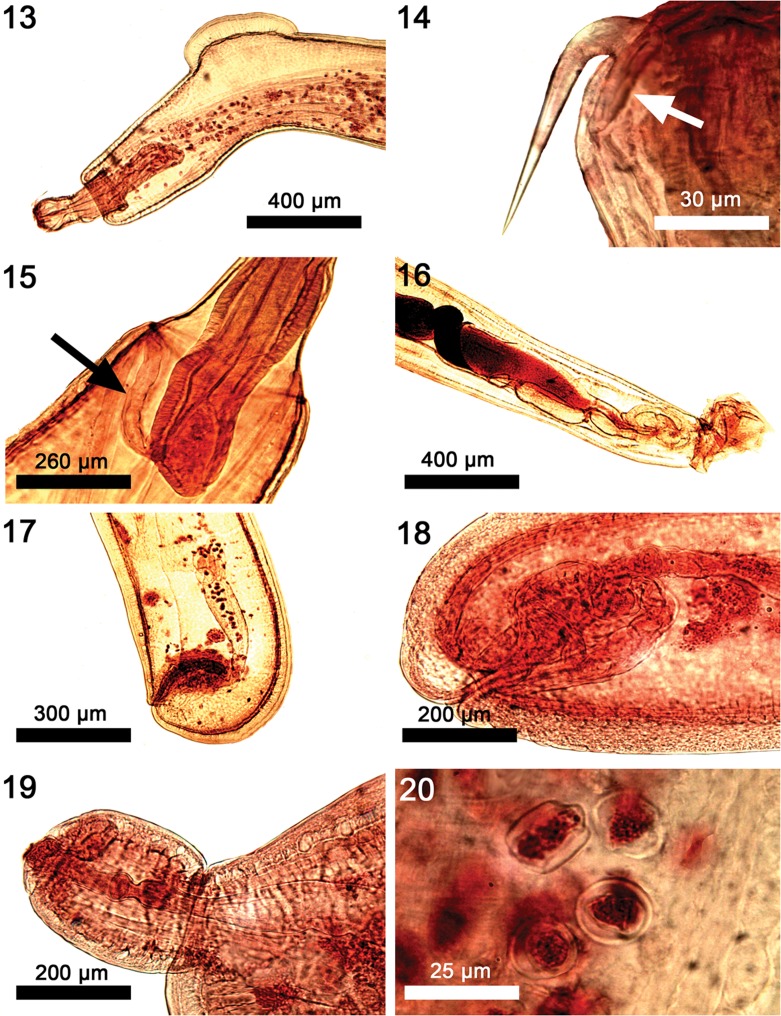
Microscopic images of specimens of *Neoechinorhynchus johnii* from *Eleutheronema tetradactylus, Johnius carouna,* and *Otolithes ruber* in the Pacific Ocean off Vietnam. (13) The anterior end of a male specimen showing the typical dorsal curvature and humping. (14) The anterior proboscis showing its characteristic curvature at the base and the prominent posteriorly directed root (arrow). (15) An anterior trunk section showing the para-receptacle structure (arrow) penetrating into the posterior receptacle wall near the large cephalic ganglion. (16) The male reproductive system of a young specimen. (17) The posterior end of a female reproductive system with a typical expanded curved vagina and a subventral gonopore. (18) Another typically convoluted posterior end of a female reproductive system. (19) An image showing an often-observed constriction at the posterior tip of a female trunk. (20) A rare image of the two types of eggs in the same frame, the spheroid egg and the lantern-like egg.

General. Neoechinorhynchidae. With characters of the genus *Neoechinorhynchus* and the subgenus *Neoechinorhynchus* as described by Amin [2]. With prominent sexual dimorphism in size of shared structures. Trunk elongate, cylindrical and slender, slightly but decidedly wider anteriorly. Body wall with osmiophilic micropores throughout (Figs. 6 and 7), often with paired cuticular folds and occasionally with dorsal hump (Fig. 13). Giant hypodermal nuclei not readily observed. Proboscis rounded but slightly wider than long (Fig. 1); with prominent nucleated apical organ. Anterior hooks long with shorter posteriorly directed spoon shaped roots, at apical end of proboscis (Figs. 2 and 14). Middle and posterior hooks small, almost equal, with short stout roots, close together and distant from anterior hooks. Middle hooks, near posterior end of proboscis; posterior hooks slightly smaller, at the attenuated posterior end of proboscis (Fig. 3). Hooks with thin cortical layer and solid but vacuolated core (Figs. 4 and 5). Neck prominent, longer than proboscis, wider at base (Figs. 1 and 15). Proboscis receptacle more than twice as long as proboscis, with ovoid cephalic ganglion at its base and para-receptacle structure (PRS) at least on one side (Fig. 15, arrow). Lemnisci long, slightly sub-equal, with two prominent ovoid giant nuclei each. Gonopore terminal in males and subterminal in females (Figs. 17 and 18).

**Males** (based on 11 adult specimens from 4 species of fish). See Tables 2 and 3 for measurements. Reproductive system in posterior half of trunk with two long tubular contiguous testes filling width of body cavity. Posterior testis longer than anterior testis. Syncytial cement gland distant from posterior testis; with many linearly arranged large spherical nuclei. Cement reservoir prominent, bulky, elongate, at posterior end of cement gland followed posteriorly by large triangular sperm vesicle, broadest anteriorly, overlapping Saefftigen’s pouch. Bursa without prominent ornamentation or marked sensory structures (Fig. 9).

**Table 2:** Morphometric comparisons between our specimens of *Neoechinorhyhchus johnii* from Vietnam and those reported in previous descriptions from Japan and the Indian Ocean.

Characters	Yamaguti (1939)	Bilqees (1972)	Gupta and Jain (1983)	Present paper
	East China Sea	Karachi coast, Pakistan	off Panaji, Bombay, India	Pacific Ocean off Vietnam
Hosts	*Johnius goma*	*Pseudosciaena diacanthus*	*Pseudosciaena diacanthus*	*Eleutheronema tetradactylum, Otolithes ruber, Johnius carouna, Johnius* sp.
Specimens described	4 females	1 male, 4 females	6 of 51 males, 0 of 35 females	11 males, 32 females
Males				
Trunk *L* × *W* (mm)	____	2.8 × 0.08	21.71–26.97 × 0.55–0.65	13.35–35.00 (25.26) × 0.35–0.75 (0.51)*
Proboscis *L* × *W*	____	80 × 80	101–142 × 129–191	120–157 (136) × 150–167 (146)
Hook from ant.	____	100, 30–40, 17–20	89–93, 20–24, 20–24	67–112 (93), 25–30 (27), 22–27 (25)
Neck *L* × *W*	____	____	142–211 × 146–215	114–281 (207) × 146–279 (204)
Prob. Receptacle *L* × *W*	____	170 × ____	406–536 × 130–163	300–572 (432) × 92–166 (135)
Long lemniscus *L* × *W*	____	ca. 1.00	1.82-2.92 × 0.08-0.11	2.24–3.12 (2.51) × 0.06–0.10 (0.08)
Short lemniscus *L* × *W*	____	ca. 1.00	1.82–2.92 × 0.08–0.11	2.27–3.12 (2.41) × 0.06–0.10 (0.07)
Ant. Testis *L* × *W* (mm)	____	Ovate 0.13 × 0.10	1.95–3.44 × 0.23–0.44	2.75–8.12 (4.80) × 0.17–0.50 (0.32)
Post. Testis *L* × *W* (mm)	____	Tubular 1.89 × 0.05	2.11–4.27 × 0.21–0.42	3.00–10.00 (5.75) × 0.20–0.62 (0.32)
Cement gland *L* × *W* (mm)	____	1.51 × 0.02	3.22–4.83 × 0.19–0.34	1.15–4.87 (3.01) × 0.12–0.34 (0.21)
Cement gland nuclei	____	____	12–13	16–25 (20.3)
Cement reservoir *L* × *W*	____	310 × 50	406–504 × 195–293	364–624 (416) × 104–281 (187)
Sperm vesicle *L* × *W*	____	250 × 50	525–1008 × 146–228	520–1144 (888) × 94–250 (177)
Saefftigen’s pouch *L* × *W*	____	____	601–780 × 81–146	593–936 (749) × 84–208 (146)
Bursa *L* × *W*	____	Papillated 50 × 100	325–861 × 162–552	364–624 (489) × 353–676 (469)
Females				
Trunk *L* × *W* (mm)	40.00–63.00 × 0.95–1.10	45.00–61.00 × 0.70–1.13	____	10.00–120.00 (44.75) × 0.27–1.17 (0.64)
Proboscis *L* × *W*	____ × 110–120	80–160 × 90–160	____	112–187 (154) × 120–182 (159)
Hook from ant.	90–100, 21–24, 21–24	80–90, 50–60, 19–20	____	62–117 (102), 25–31 (27), 22-30 (26)
Neck *L* × *W*	170–250 × 110–130	208–302 (246) × 146–281 (196)	____	
Prob. Receptacle *L* × *W*	400–520 × 90–140	38 × ____	____	255–551 (466) × 87–177 (129)
Long lemniscus *L* × *W*	2.50–3.80 × 0.10–0.11	2.70–3.30 × ____	____	2.39–3.33 (2.87) × 0.006–0.18 (0.09)
Short lemniscus *L* × *W*	2.50–3.80 × 0.10–0.11	2.70–3.30 × ____	____	2.39–3.17 (2.73) × 0.06–0.15 (0.08)
Vagina length	____	200–250	____	156–260 (209)
Uterus length	250–480 × 80–125	300–400	____	250–572 (454)
Uterine bell length	____	140–170 × 100–160	____	250–624 (477)
Reprod. syst. *L* (mm)	____	____	____	0.67–1.51 (1.13)
Egg *L* × *W*	33 × 18	29–30 × 20	33–38 × 20–22	27–45 (33) × 18–30 (24)

* Range (mean).

**Table 3:** The relationship between host species and size of certain anatomical structures of 43 measured specimens of *Neoechinorhynchus johnii* collected off the Pacific coast of Vietnam mostly in 2017.

Worm sex	Character	Host species		
		*Eleutheronema tetradactylum* (*n* = 13)	*Johnius carouna* (*n* = 22)	*Otolithes ruber* (*n* = 8)
Female	Trunk length (mm)	15.0–120.00 (62.58)*	10.00–46.25 (31.77)	50.00–61.00 (55.00)
Male	Trunk length (mm)	25.60	13.75–27.50 (22.50)	30.00–35.00 (32.00)
Female	Proboscis length	112–145 (131)	142–162 (151)	160–187 (169)
Female	Ant. Proboscis hook *L*	62–100 (83)	97–117 (107)	97–112 (109)
Male	Ant. Proboscis hook *L*	62–67 (64)	92–112 (104)	107
Female	Prob. Receptacle *L*	255–468 (380)	400–551 (475)	510–551 (530)
Male	Prob. Receptacle *L*	343	300–468 (401)	572
Male	Post. Testis *L* (mm)	4.25	3.00–10.00 (5.25)	8.12–8.95 (8.53)

* Range (mean).

**Females** (based on 32 adults with eggs and ovarian balls from 4 species of fish). See Tables 2 and 3 for measurements. Reproductive system with prominent vaginal sphincter, bulb and tube, thick-walled fusiform uterus, few large uterine glands of selector apparatus, and large conical uterine bell attached to body wall ventrally. Adults with introverted genital vestibule lined with invaginated body wall (Figs. 17 and 18) but occasionally adults and immatures with terminal or subterminal digitiform prolongation of posterior trunk tip with terminal gonopore (Figs. 11 and 19). Eggs either spherical and thick walled showing no prolongation of fertilization membrane or more rectangular with soft corners and two cone-shaped poles (Figs. 12 and 20).

### Relationships to host species

The measurements provided for specimens of *N. johnii* collected from four species of fish (Table 2) demonstrate considerable variability in the size of certain structures that appear to be associated with host species (Table 3). Most noticeably, the longest worms from *E. tetradactylum* (females reaching 120.0 mm in length) had the shortest anterior proboscis hooks in males and females averaging 64–83 long compared to anterior hooks of worms from *J. carouna* and *O. ruber* that exceeded 100 in length. Size of the proboscis, proboscis receptacle, and testes was similarly smaller in worms obtained from *E. tetradactylum* compared with worms from *J. carouna* and *O. ruber*.

### Remarks

We have provided the morphological description of 11 male and 32 female specimens of *N. johnii* from 20 and 40 specimens obtained from four new species of fish collected along the Pacific coast of Vietnam (Table 1). The previous three descriptions were incomplete and inadequate. Yamaguti [40] originally described the species from four females with two line drawings (his Figs. 37 and 38) collected from *Johnius goma* (Tanaka) in the East China Sea. Bilqees [21] described one 2.8 mm long male of uncertain identity with ovate anterior testis and four females with line drawings (her Figs. 9–16) from *Pseudosciaena diacanthus* Lacepède off the Karachi coast of Pakistan. Gupta and Jain [25] described only six males from *D. diacanthus* also in the Indian Ocean off Panaji and Bombay, India, even though they collected 51 males and 35 females but provided line drawings of both males and females (their Fig. 2a–h). Bilqees [21] reported unusually large middle proboscis hooks, two or three times as large as the posterior hooks, which is a serious departure from the original description of the species where middle and posterior hooks have the same size. Gupta and Jain [25] mentioned egg size in females that were not described. The size of most structures of the single diminutive male from Pakistan [21] could not be objectively compared to that of corresponding structures from the six males from India [25] collected from the same host species, *P. diacanthus*, to examine the influence of host species. Comparative measurements in Table 2 show that our specimens from four host species in Vietnam collectively had the largest size of trunk, proboscis, proboscis hooks, neck, and testes, and more cement gland nuclei. When those measurements were broken down by host species (Table 3), the relationship of host species to the size of taxonomically important structures such as trunk, anterior proboscis hooks, proboscis and testes became quite apparent.

We consider the line drawings of the above authors sufficient to give a sense of the morphology of *N. johnii* and we provide additional insights and new information of various anatomical structures of this species using photo-microscopy (Figs. 13–20) and SEM (Figs. 1–12) of our specimens collected off the Pacific coast of Vietnam (Table 1). Our Vietnamese hosts are of Indo-West Pacific distribution extending between India and east Africa to the west and the Gulf of Thailand, southern China, and the West Pacific to the east [32, 35]. Our findings provide the first complete description of this species and show considerable variations between our Vietnamese specimens and those reported by other authors (Table 2). Our findings also demonstrate the relationships between size of certain structures and host species (Table 3).

### EDAX findings

**Figure 21:**
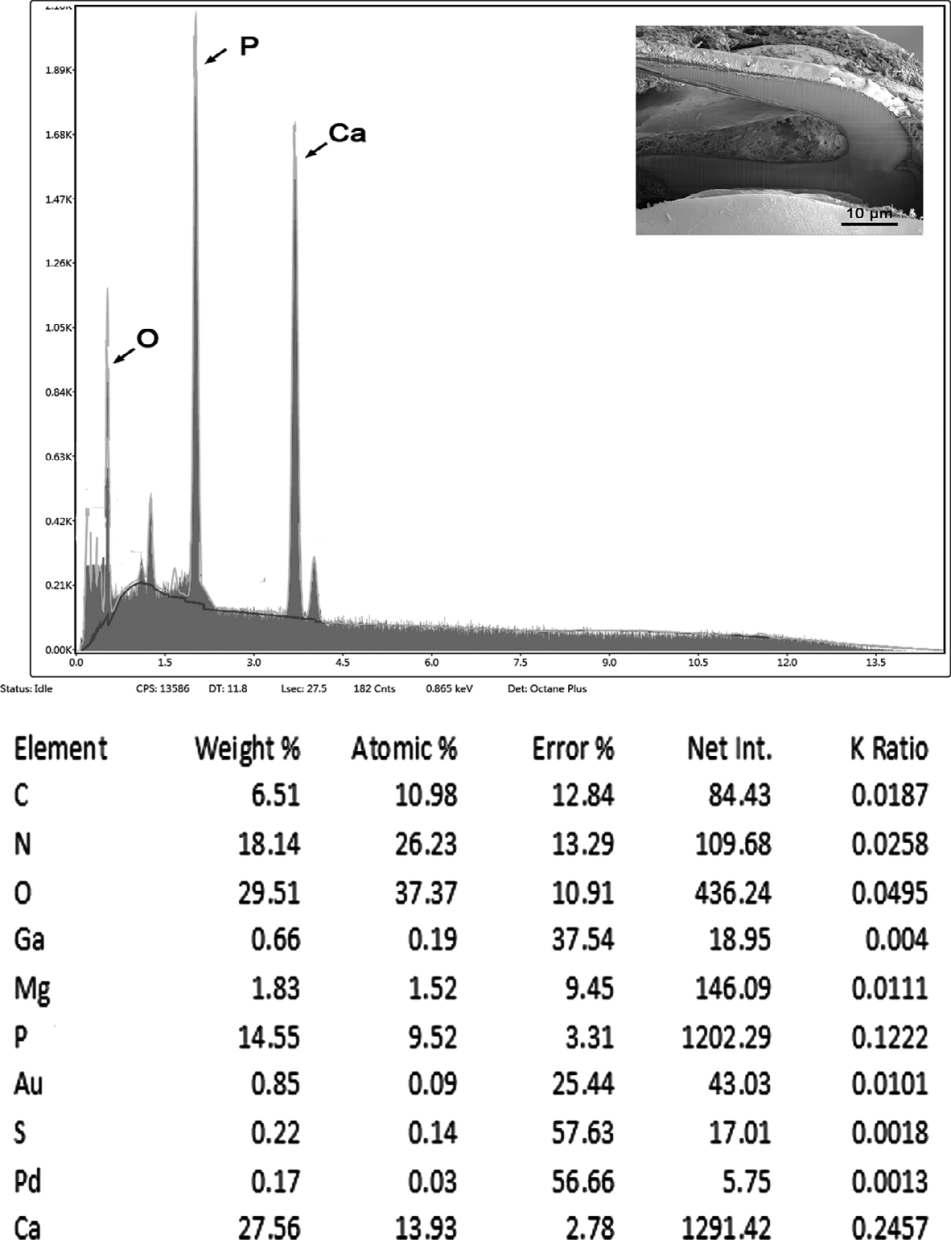
Energy Dispersive X-Ray spectrum of the base center of a large hook of a *Neoechinorhynchus johnii* specimen showing high levels of calcium and phosphorus (see Table 4). Insert: SEM of a lateral longitudinal gallium cut hook. The X-ray data are the elemental analysis of the hook base.

Table 4 and Figure 21 show the results of the hook chemical element analysis. Chemical elements observed were typical for acanthocephalan hooks with high sulfur content on the outer edge of the hook and increased amounts of calcium and phosphorus in the center areas. Both cross-section cuts and longitudinal cuts were analyzed.

**Table 4:** Chemical composition of a Gallium (Ga, LMIS) cut of anterior hook of *Neoechinorhynchus johnii.*

Element***	Hook tip*		Hook middle*		Base of hook**	
	Edge	Center	Edge	Center	Arch	Bottom
Magnesium (Mg)	0.07	1.25	0.57	2.21	1.83****	2.05
Aluminum (Al)	0.31	0.27	0.42	0.1	**0**	0
Phosphorous (P)	1.62	10.69	3.62	14.32	**14.55**	15.51
Sulphur (S)	15.57	5.14	11.73	0.61	**0.22**	0.23
Calcium (Ca)	2.36	19.84	4.66	26.79	**27.56**	31.12

* Cross section cut.

** Longitudinal cut.

*** Common protoplasmic elements (C, N, O) and processing elements (Au, Pd, Ga) omitted from table. Listed in wt%.

**** Bold weight % figures are used to generate the spectrum (Fig. 21).

### Molecular description

**Figure 22:**
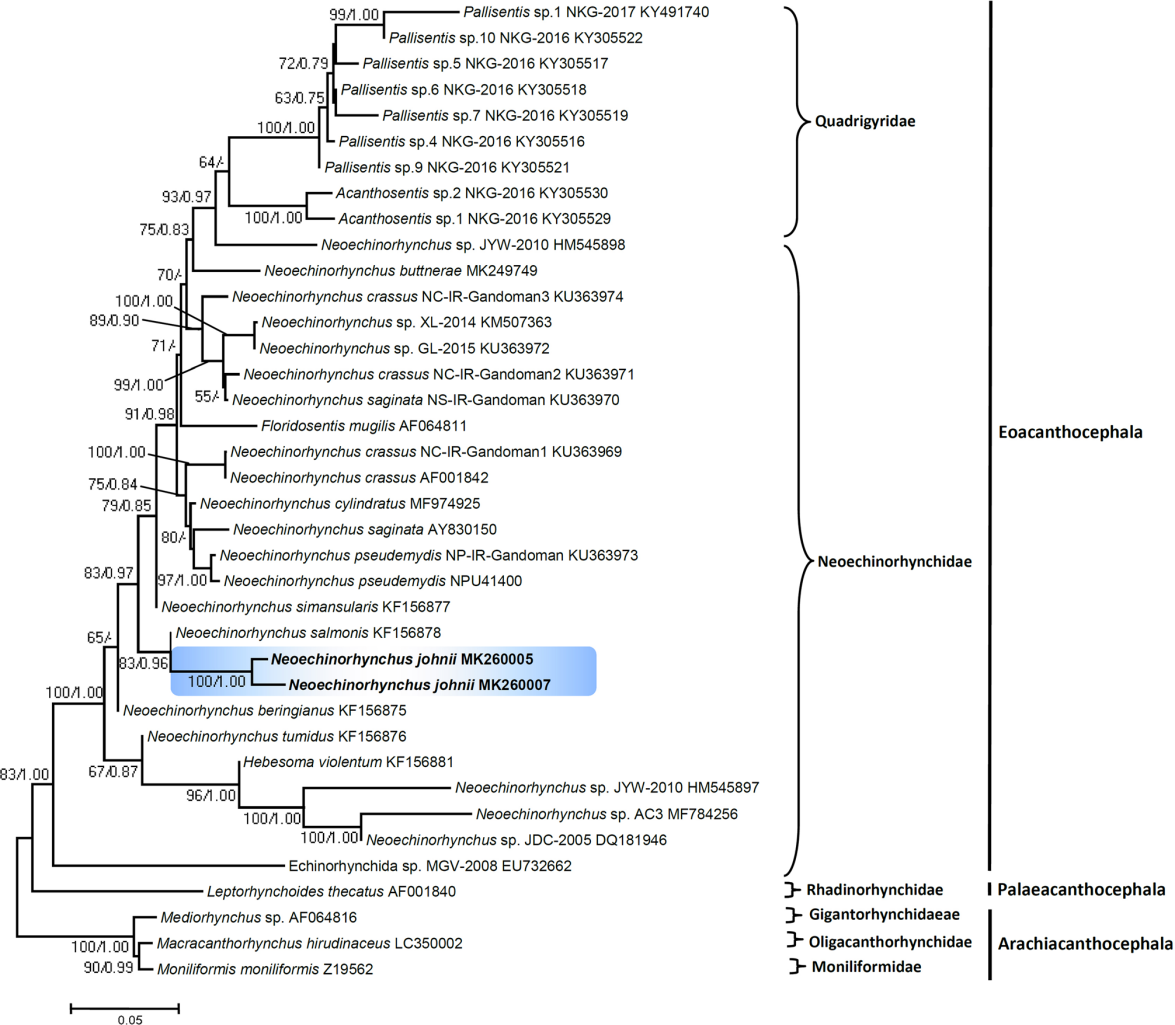
Phylogenetic tree of *Neoechinorhynchus johnii* species of the nucleotide 18S data set. The bootstrap values are listed in the order: ML/BI. Hyphen indicates a node unsupported by BI. GenBank accession numbers are provided alongside the species names. Species of Archiacanthocephala were selected as the out-group species for the 18S gene.

**Figure 23:**
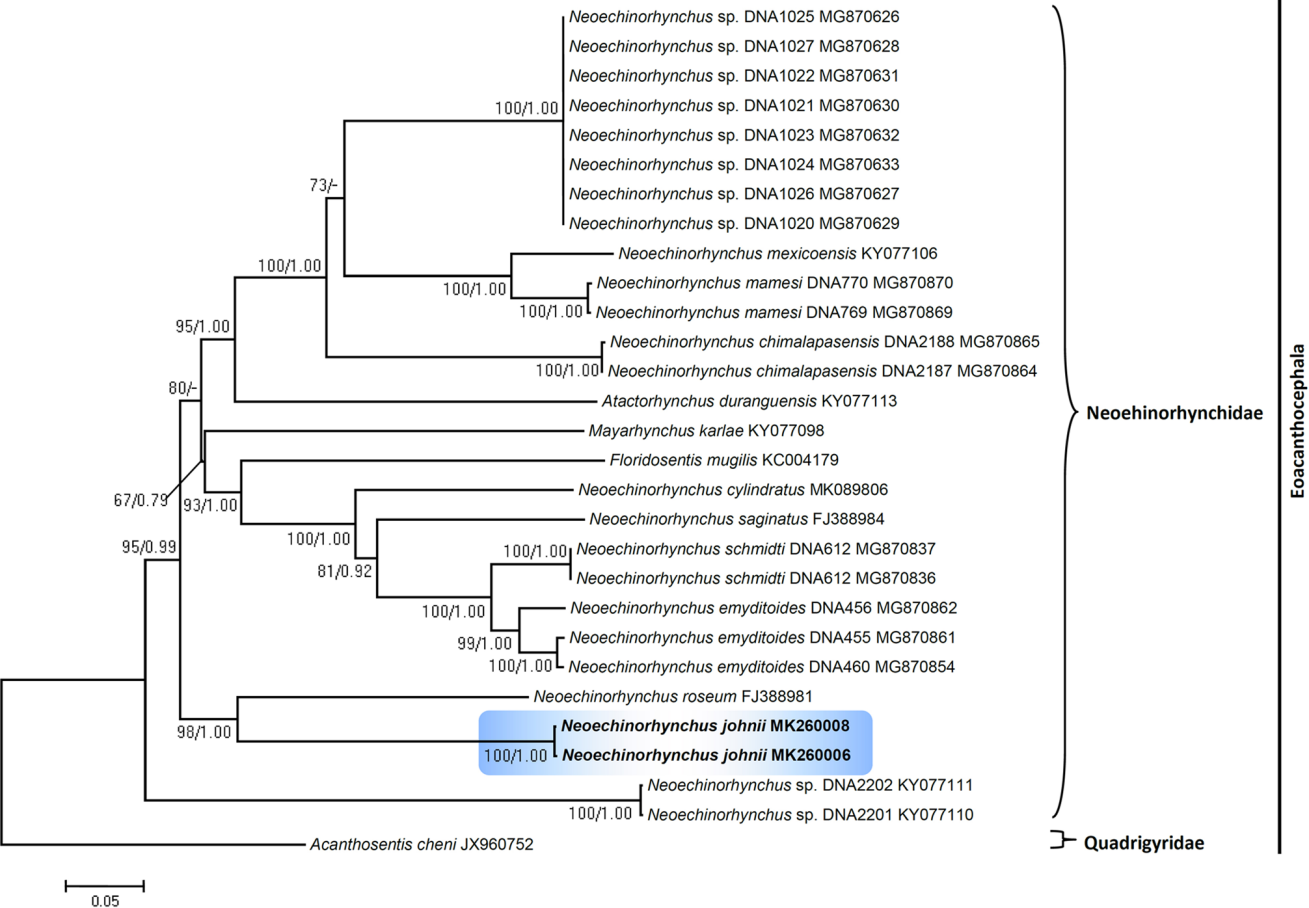
Phylogenetic analysis of the ribosomal ITS1-5.8S-ITS2 region using the maximum likelihood method. Numbers at nodes indicate ML bootstrap values (1000 replications) and posterior probabilities (BI), respectively with GenBank accession numbers listed alongside the species names. Hyphen indicates a node unsupported by BI. *Acanthosentis cheni* (JX960752) was selected as the out-group species for the ITS1+5.8S+ITS2 region.

In this study, we compared DNA sequences of *N. johnii* species from Vietnam with sequences of the closely related species of the same genus, *Neoechinorhynchus*, and other acanthocephalans retrieved from GenBank (Tables 5 and 6). For the analysis, we choose the 18S gene as it is highly conserved and better for an analysis of the upper level phylogeny, while the ITS1-5.8S-ITS2 region is additionally beneficial to differentiate between species. DNA fragments of the 18S and ITS1-5.8S-ITS2 datasets were sequenced for individuals of *N. johnii.* Our sequences from two isolates from two different hosts of *N. johnii* for 18S and the ITS1+5.8S+ITS2 region show no detectable intraspecific sequence variability among the individuals sampled. Isolates from all host species were not possible because of limited sample sizes. The genetic divergence estimated among the species of *Neoechinorhynchus* used for phylogenetic analysis (Table 5 and 6) ranged from 0.06 to 0.28% for 18S and from 0.15 to 0.24% for the ITS1-5.8S-ITS2 region, respectively. For the 18S gene, both ML and BI analyses recovered the newly generated sequence for *N. johnii* from Vietnam and formed a strongly supported individual sister clade (100/1.00). The isolate of *N. johnii* obtained demonstrated the association with *Neoechinorhynchus salmonis* Ching, 1984 (KF156878) from Lake Chistoe, Russia. Both the species show 92.86% identity along with other species of *Neoechinorhynchus* from a clade belonging to Mexico, China, Iran, Russia and the USA (Fig. 22). For the ITS1+5.8S+ITS2 sequence dataset, the phylogenetic analyses resulting from both ML and BI analyses were highly congruent. Newly generated sequences for *N. johnii* clustered together with the sequences of *N. roseum* Salgado-Maldonado, 1978 (FJ388981) from Mexico were strongly associated (98/1.00%) with other species of *Neoechinorhynchus* from Mexico (Fig. 23). When the data set consisted of 18S and the ITS1-5.8S-ITS2 region used to generate the tree, the tree showed paraphyly of the genus *Neoechinorhynchus* that can be studied more congruently after addition of other congeneric species data worldwide.

**Table 5:** Acanthocephala species, origin and GenBank accession numbers used for phylogenetic analysis based on the 18S region. Sequences marked with an asterisk were obtained in this study. Na = not available.

Species	Host	Host origin	GenBank accession no.
***Neoechinorhynchus* Stiles and Hassall 1905**			
*Neoechinorhynchus tumidus*	*Coregonus nasus*	Russia	KF156876
*Neoechinorhynchus simansularis*	*Salvelinus alpinus*	Russia	KF156877
*Neoechinorhynchus salmonis*	*Onchorynchus nerka*	Russia	KF156878
*Neoechinorhynchus saginata*	Na	USA	AY830150
*Neoechinorhynchus beringianus*	*Pungitius pungitius*	Russia	KF156875
*Neoechinorhynchus pseudemydis*	*Capoeta aculeata*	Iran	KU363973
*Neoechinorhynchus pseudemydis*	Na	USA	NPU41400
*Neoechinorhynchus crassus*	Na	Iran	KU363969
*Neoechinorhynchus crassus*	Na	USA	AF001842
*Neoechinorhynchus* sp.	Na	China	KM507363
*Neoechinorhynchus* sp.	*Capoeta aculeata*	Iran	KU363972
*Neoechinorhynchus saginata*	Na	Iran	KU363970
*Neoechinorhynchus crassus*	*Capoeta aculeata*	Iran	KU363974
*Neoechinorhynchus crassus*	*Capoeta aculeata*	Iran	KU363971
*Neoechinorhynchus* sp.	*Siganus fuscescens*	China	HM545898
*Neoechinorhynchus buttnerae*	Na	Brazil	MK249749
*Neoechinorhynchus cylindratus*	*Micropterus salmoides*	USA	MF974925
*Neoechinorhynchus johnii*	*Eleutheronema tetradactylus*	Vietnam	MK260005*
*Neoechinorhynchus johnii*	*Johnius carouna*	Vietnam	MK260007*
*Neoechinorhynchus* sp.	*Siganus fuscescens*	China	HM545897
*Neoechinorhynchus* sp.	*Heteropneustes fossilis*	India	MF784256
*Neoechinorhynchus* sp.	*Oreochromis niloticus*	Democratic Republic of the Congo	DQ181946
***Pallisentis* Van Cleave 1928**			
*Pallisentis* sp. 1	Na	India	KY491740
*Pallisentis* sp. 4	Na	India	KY305516
*Pallisentis* sp. 5	Na	India	KY305517
*Pallisentis* sp. 6	Na	India	KY305518
*Pallisentis* sp. 7	Na	India	KY305519
*Pallisentis* sp. 9	Na	India	KY305521
*Pallisentis* sp. 10	Na	India	KY305522
***Acanthosentis* Verma and Datta 1929**			
*Acanthosentis* sp. 1	Na	India	KY305529
*Acanthosentis* sp. 2	Na	India	KY305530
***Floridosentis* Ward 1953**			
*Floridosentis mugilis*	*Mugil cephalus*	Mexico	AF064811
***Hebesoma* Van Cleave 1928**			
*Hebesoma violentum*	*Perccottus glenii*	Russia	KF156881
***Echinorhynchidae* (Ward, 1917) Van Cleave, 1928**			
Echinorhynchida sp.	*Cichlasoma urophthalmus*	Mexico	EU732662
***Leptorhynchoides* Kostylev 1924**			
*Leptorhynchoides thecatus*	*Lepomis cyanellus*	USA	AF001840
**Outgroup**			
*Moniliformis moniliformis*	Na	UK	Z19562
*Macracanthorhynchus hirudinaceus*	*Sus scrofa leucomystax*	Japan	LC350002
*Mediorhynchus* sp.	*Casidix mexicanus*	Mexico	AF064816

**Table 6:** Acanthocephala species, origin and GenBank accession numbers used for phylogenetic analysis based on the ITS1-5.8S-ITS2 gene cluster. Sequences marked with an asterisk were obtained in this study. Na = not available.

Species	Host	Host origin	GenBank accession no.
***Neoechinorhynchus* Stiles and Hassall 1905**			
*Neoechinorhynchus* sp.	*Dormitator latifrons*	Mexico	MG870633
*Neoechinorhynchus* sp.	*Dormitator latifrons*	Mexico	MG870632
*Neoechinorhynchus* sp.	*Dormitator latifrons*	Mexico	MG870630
*Neoechinorhynchus* sp.	*Dormitator latifrons*	Mexico	MG870631
*Neoechinorhynchus* sp.	*Dormitator latifrons*	Mexico	MG870626
*Neoechinorhynchus* sp.	*Dormitator latifrons*	Mexico	MG870627
*Neoechinorhynchus* sp.	*Dormitator latifrons*	Mexico	MG870628
*Neoechinorhynchus* sp.	*Dormitator latifrons*	Mexico	MG870629
*Neoechinorhynchus mamesi*	*Dormitator latifrons*	Mexico	MG870870
*Neoechinorhynchus* sp.	Na	Mexico	KY077110
*Neoechinorhynchus* sp.	Na	Mexico	KY077111
*Neoechinorhynchus roseum*	*Citharichthys gilbertei*	Mexico	FJ388981
*Neoechinorhynchus mamesi*	*Dormitator latifrons*	Mexico	MG870869
*Neoechinorhynchus chimalapasensis*	*Awaous banana*	Mexico	MG870865
*Neoechinorhynchus chimalapasensis*	*Awaous banana*	Mexico	MG870864
*Neoechinorhynchus johnii*	*Eleutheronema tetradactylum*	Vietnam	MK260008*
*Neoechinorhynchus johnii*	*Johnius carouna*	Vietnam	MK260006*
*Neoechinorhynchus schmidti*	*Trachemys scripta*	Mexico	MG870837
*Neoechinorhynchus schmidti*	*Trachemys scripta*	Mexico	MG870836
*Neoechinorhynchus emyditoides*	*Trachemys scripta*	Mexico	MG870862
*Neoechinorhynchus emyditoides*	*Trachemys scripta*	Mexico	MG870861
*Neoechinorhynchus emyditoides*	*Trachemys scripta*	Mexico	MG870854
*Neoechinorhynchus mexicoensis*	*Dormitator maculatus*	Mexico	KY077106
*Neoechinorhynchus cylindratus*	*Micropterus salmoides*	Mexico	MK089806
*Neoechinorhynchus saginatus*	NA	Mexico	FJ388984
***Atactorhynchus* Chandler 1935**			
*Atactorhynchus duranguensis*	*Cyprinodon meeki*	Mexico	KY077113
***Floridosentis* Ward 1953**			
*Floridosentis mugilis*	*Mugil cephalus*	Mexico	KC004179
***Mayarhynchus* Pinacho-Pinacho, Hernández-Orts, Sereno-Uribe, Pérez-Ponce de León and García-Varela 2017**			
*Mayarhynchus karlae*	*Thorichthys ellioti*	Mexico	KY077098
**Outgroup**			
*Acanthosentis cheni*	*Coilia nasus*	China	JX960752

In spite of the increasing number of described species of acanthocephalans from Vietnam, no molecular data are available for species of the genus *Neoechinorhynchus* to date. Molecular sampling of members of the genus *Neoechinorhynchus* is still lacking. The 18S and ITS1-5.8-ITS2 datasets included in our study are the only representative sequences for *N. johnii* to date.

## Discussion

We provide a complete description of *N. johnii* for the first time from four new species of fish off the Vietnam Pacific coast. Our material fills certain serious gaps in knowledge of the species, which was based on previous incomplete and inadequate descriptions. This acanthocephalan species is clearly of Indo-Pacific distribution and is found in hosts that naturally inhabit waters off the Japanese islands, India and Pakistan. Its distribution in the Pacific waters of Vietnam extended from Tien Yen in the north to Vung Tau, Nha Trang, and Bac Lieu in the south (Table 1). It will not be surprising to find this acanthocephalan from the same host species elsewhere within their endemic range in the Indo-Pacific region.

### Variability

Much of the reported variability in the size of taxonomically important structures such as the trunk, proboscis hooks, proboscis, testes, etc. has been attributed to host species. Such relationships have previously been reported in other species of acanthocephalans including *Echinorhynchus salmonis* Müller, 1784 in Lake Michigan where male and female specimens from bloater, *Coregonus hoyi* (Gill) (Salmonidae) achieved not only larger size but also different body form (broad anteriorly) compared to the slender specimens from rainbow smelt, *Osmerus mordax* (Mitchell) (Osmeridae) [4]. The larger and heavier worms from bloater almost invariably showed higher regression coefficients (adjusted coefficient of determination) compared to those from smelt in all characters including size of trunk, proboscis, longest proboscis hooks, receptacle, testes, lemnisci, and eggs. The taxonomic implications of this variability were discussed [4], but the reported material from Vietnam remains attributable to *N. johnii*.

Earlier, Amin [1] demonstrated a similar relationship for *Acanthocephalus dirus* (Van Cleave, 1931) Van Cleave and Townsend, 1936 in Wisconsin fishes. Females of the same developmental stage recovered during the same period were found to have attained larger sizes in certain hosts than in others, with the largest females being found in *Lepomis macrochirus* Rafinesque. The size of the trunk in males was also found to follow the same pattern. Similarly, testes also attained a larger size in males recovered from *Catostomus commersonii* Lacépède (Catostomidae) than in males from *Semotilus atromaculatus* (Mitchill) (Cyprinidae). Amin [1] stated that these size variations “result from differential growth rates of these worms in the various host intestinal environments (and) are probably mediated by certain host specific factors.”

### The para-receptacle structure (PRS)

The PRS inserts anteriorly in the body wall near the neck and posteriorly at the posterior end of the receptacle. The presence of the PRS in eoacanthocephalans with a weak single proboscis receptacle wall was first demonstrated in *Neoechinorhynchus* (*N*.) *qatarensis* Amin, Saoud, Alkuwari, 2002 [19] and has since been reported in other species of *Neoechinorhynchus* and *Acanthogyrus* (*Acanthosentis*) Verma and Datta, 1929 reviewed in part in Amin et al. [15] and reported here for the first time in various species of *Neoechinorhynchus* from marine fishes off the east coast of Vietnam. In the description of the PRS, Amin et al. [7, 19] proposed that it may regulate the hydrostatic pressure in the receptacle to facilitate the retraction and eversion of the proboscis.

### Electron dense micropores

Micropores are present throughout the epidermal surface of the trunk of reported species of *Neoechinorhynchus*, like those reported in other species of the Acanthocephala, and are associated with internal crypts and vary in diameter and distribution in different trunk regions, corresponding to differential absorption of nutrients. We have documented this phenomenon in 16 species of acanthocephalans [27]. The functional aspects of micropores in a few other acanthocephalan species including *Rhadinorhynchus ornatus* Van Cleave, 1918, *Polymorphus minutus* (Goeze, 1782) Lühe, 1911, *Moniliformis* (Bremser, 1811) Travassos (1915), *Macracanthorhynchus hirudinaceus* (Pallas, 1781) Travassos (1916, 1917), and *Sclerocollum rubrimaris* Schmidt and Paperna, 1978 were reviewed earlier [8]. We demonstrated the tunneling from the cuticular surface into the internal crypts by TEM. Wright and Lumsden [39] and Byram and Fisher [22] reported that the peripheral canals of the micropores are continuous with canalicular crypts. These crypts appear to “constitute a huge increase in external surface area implicated in nutrient uptake.” Whitfield [38] estimated a 44-fold increase at a surface density of 15 invaginations per 1 μm^2^ of *Moniliformis moniliformis* (Bremser, 1811) Travassos, 1915 tegumental surface. The micropores and the peripheral canal connections to the canaliculi of the inner layer of the tegument of *Corynosoma strumosum* (Rudolphi, 1802) Lühe, 1904 from the Caspian seal *Pusa caspica* (Gmelin) in the Caspian Sea were demonstrated by transmission electron micrographs [17].

### EDAX of hooks

Most acanthocephalan hooks have three major elements: calcium, phosphorus, and sulfur [15, 16], among others, and small amounts of magnesium plus common protoplasm elements (C, N, O, H). The arched area at the base of the anterior hook of *N. johnii* exhibited high levels of calcium and phosphorus (Table 4, Fig. 21) very similar to the mammalian tooth with its layers. The X-ray scans of the gallium cut hooks help explain the morphological nature of *N. johnii* and identify its unique “personality.” The uniqueness of the metal analysis as expressed by X-ray scans appear to be species-specific and can be regarded as finger prints of key diagnostic value that are just as important as molecular analysis.

### Phylogenetic analysis

Results consistently yielded a strongly supported distinct clade for the *Neoechinorhynchus* species from Vietnam for both 18S and the ITS1-5.8-ITS2 region of ribosomal RNA. Phylogenetic analysis demonstrated that *N. johnii* occupies a separate position in the trees, probably indicating an Asian origin of this species. It has already been described in various studies that the genus *Neoechinorhynchus* harbors rich species diversity [3, 33, 34], but the lack of molecular data from the Asian region for species of *Neoechinorhynchus* demonstrates that the sampling of members of the genus, especially in Asia, is still incomplete. Previous molecular studies also reported paraphyly within the Palaeacanthocephala [28, 37], while findings related to monophyly in Eoacanthocephala are yet to be elucidated. Considering the results of the present study and the incompleteness of taxon sampling for species of *Neoechinorhynchus,* it would be very early to draw any conclusions regarding reconstruction of the evolutionary history of this group of acanthocephalans. Regarding the monophyletic and/or paraphyletic assemblage of *N. johnii*, we found that the sequences were not construed as a monophyletic assemblage in both regions (18S and ITS1-5.8S-ITS2) analyses. The present analyses show that the genus is paraphyletic and our phylogenetic trees corroborate the findings of other researchers [24, 34]. Thus, the genus *Neoechinorhynchus* requires more investigation as it has rich species diversity and, most likely, does not represent a monophyletic group. Therefore, this genus requires taxonomic revision with generation of more molecular data from additional species that will help to understand the phylogenetic relationships more clearly. In conclusion, our findings emphasize the importance of using both morphological and molecular methods when evaluating acanthocephalan diversity.
